# Bone homeostasis disorders increased the mortality of sepsis patients: A preliminary retrospective cohort study

**DOI:** 10.3389/fmed.2022.1017411

**Published:** 2022-12-01

**Authors:** Dong Wang, Jingyi Wang, Xi Zheng, Shuo Diao, Wenxiong Li, Wenliang Ma

**Affiliations:** ^1^Department of Orthopedics, Beijing Chaoyang Hospital, Capital Medical University, Beijing, China; ^2^Department of SICU, Beijing Chaoyang Hospital, Capital Medical University, Beijing, China

**Keywords:** bone homeostasis disorders, CTX-I, TRACP-5b, PIEZO1, sepsis, sepsis shock, mortality, retrospective cohort study

## Abstract

**Introduction:**

Sepsis is a common clinical syndrome and nearly 20% of all deaths are related to sepsis. As an important part of the body, bone homeostasis disorders are closely related to inflammatory response, but the correlation between bone homeostasis and sepsis, sepsis shock was unknown. The objective of this study was to explore the relation of bone homeostasis on sepsis and sepsis shock.

**Methods:**

In this retrospective cohort study, patients were enrolled between April 2018 and May 2022 from Beijing Chaoyang hospital. Primary outcomes were serum indicators reflected bone homeostasis, such as cross-linked carboxy-terminal telopeptide of type I collagen (CTX-I), tartrate-resistant acid phosphatase 5b (TRACP-5b) and piezo-type mechanosensitive ion channel component 1 (PIEZO1).

**Results:**

The data were analyzed retrospectively. among 88 evaluable patients, 45 were sepsis (19 were sepsis shock) and 43 were non-sepsis. There was no significant difference in age, gender, BMI, combination diseases, operation time, intraoperative blood loss, and hospital stay. Patients with sepsis or sepsis shock had higher serum CTX-I, TRACP-5b, PIEZO1 (*p* < 0.05). Spearman’s rank correlation test showed that CTX-I, TRACP-5b, PIEZO1 and the three together (CTX-I + TRACP-5b + PIEZO1) had strong correlation with sepsis or sepsis shock (*p* < 0.05). The receiver operating characteristic curve (ROC) and precision-recall curve (PRC) showed that these indicators could predict the occurrence of sepsis or sepsis shock (*p* < 0.05). Besides, decision curve analysis (DCA) and interventions avoided curve (IAC) displayed a high net benefit of bone homeostasis disorders indicators on sepsis or sepsis shock. Kaplan–Meier survival curves revealed that sepsis or shock patients with high value indicators (>0.47227) had a higher mortality (*p* < 0.05).

**Conclusion:**

Bone homeostasis disorders could increase the mortality of sepsis and sepsis shock patients.

## Introduction

Sepsis is a life-threatening organ dysfunction caused by a dysregulated host response to infection and nearly 20% of all deaths are related to sepsis (1–3). According to sepsis 3.0 diagnostic criteria, septic shock is a subtype of sepsis with more unstable circulation and higher mortality (4–8). The severity of sepsis is closely related to the body’s response to inflammation. Sepsis patients have multiple system organs regulation disorders and even function suppression (9, 10).

Bone is a tissue rich in nerve and blood vessels (11–13). Current studies have found that bone tissue is closely related to the inflammation regulation of patients (14–16). The concept of immune-skeletal interface has been proposed that immune cells directly regulate the bone microenvironment homeostasis (14, 17). Besides, osteoblast and osteoclast also play a very important role in immune cells functions and differentiation (16, 17). Although there are many studies on bone homeostasis and inflammation reaction, there is no report on the relationship between bone homeostasis and sepsis, especially sepsis shock.

We hypothesized that bone homeostasis disorders were closely related to sepsis and sepsis shock. Besides, bone homeostasis disorders would aggravate sepsis and even increase the mortality of patients. Therefore, a preliminary retrospective cohort study was conducted to investigate the relationship between bone homeostasis disorders and sepsis.

## Materials and methods

### Study design

This study was a retrospective cohort study observed the relation of bone homeostasis, sepsis and patients’ mortality. Primary outcome was serum indicators reflected bone homeostasis, such as cross-linked carboxy-terminal telopeptide of type I collagen (CTX-I), tartrate-resistant acid phosphatase 5b (TRACP-5b) and piezo-type mechanosensitive ion channel component 1 (PIEZO1). In additions, the secondary outcomes were acute physiology and chronic health evaluation-II (APACHE II) scores, sequential organ failure assessment (SOFA) scores, Combination diseases, such as hypertension, diabetes mellitus, coronary heart disease, chronic obstructive pulmonary disease (COPD), chronic kidney disease (CKD), cerebrovascular disease (CVD), acute respiratory distress syndrome (ARDS), acute kidney injury (AKI), hepatic failure, disseminated intravascular coagulation (DIC), operation received, operation time, intraoperative blood loss (IBL), intraoperative fluid balance, mechanical ventilation time, ICU stay time, hospital stay time and 30-day all caused mortality.

Besides, some blood indicators were also measured, such as blood PH value, PaCO2, HCO3- concentration, blood lactic acid concentration, white blood cell number, hemoglobin, serum creatinine, serum alanine aminotransferase (ALT), aspartate transaminase (AST), B-type natriuretic peptide (BNP), albumin, blood calcium, and blood phosphorus.

The study was performed at Beijing Chao-Yang hospital affiliated to Capital Medical University between April 2018 and May 2022. It was approved by the ethics committee of Beijing Chao-Yang Hospital Affiliated to Capital Medical University (2020-ke-236) and the data were analyzed retrospectively.

### Patients

The inclusion criteria for patients was as follows: (1) age ≥ 18 years; and (2) admitted to ICU. The exclusion criteria were: (1) pregnant and lying-in woman; (2) patients with nerve, brain or bone injury; (3) patients with metabolic and immune diseases, such as systemic lupus erythematosus, polymyositis and Sjogren’s syndrome; (4) patients treated with long-term glucocorticoid therapy; (5) patients receiving bone injury surgery, such as craniotomy, sternotomy, thoracoplasty; (6) patients with bone tumors, such as osteosarcoma, osteochondroma, bone metastasis; (7) patients with osteoarthritis, rheumatoid arthritis, bone non-union or fracture healing stage; (8) patients with insufficient serum samples and unable to complete the detection of serum bone homeostasis indicators; (9) patients loss of visit; (10) patients who did not meet the inclusion criteria.

Two clinical observers (XZ and JW) with clinical research experience enrolled the patients strictly according to the inclusion and exclusion criteria. The baseline information of patients was recorded, such as patient’s name, gender, age, and BMI.

### Sample size

Stata/MP 16.0 software was used to calculate the sample size. There is no relevant clinical study on the relationship of bone homeostasis and sepsis. Therefore, the relevant pre-experiment was carried out and two patients were enrolled. The serum PIEZO1 concentration of sepsis patients were 15.43, 18.17 ng/ml and non-sepsis patients were 13.46, 10.41 ng/ml. CTX-I concentration of sepsis patients were 5.67, 4.71 ng/ml and non-sepsis patients were 3.47, 2.51 ng/ml. TRACP-5b of sepsis patients were 4.24, 5.64 mIU/ml and non-sepsis patients were 2.58, 2.25 mIU/ml. Alpha was set as 0.01 (two-sided) and power was set as 0.95. The ratio of sepsis group and non-sepsis group was 1. The calculated minimum sample size of each group was 9.

### Definition and outcomes

Sepsis is defined by sepsis 3.0 as life-threatening organ dysfunction caused by a dysregulated host response to infection and sepsis shock defined as a subset of sepsis in which particularly profound circulatory, cellular, and metabolic abnormalities are associated with a greater risk of mortality than with sepsis alone (4). Clinical parameters to identify patients with sepsis are increasing in SOFA score ≥ 2 from baseline or qSOFA ≥ 2 and suspected infection (4, 5). The septic shock clinical parameters are: vasopressor requirement to maintain a mean arterial pressure of 65 mm Hg or greater and serum lactate level greater than 2 mmol/L (>18 mg/dl) in the absence of hypovolemia (4, 5).

The main collagen component in bone tissue is type I collagen, accounting for more than 90% of the bone matrix. CTX-I is a degradation product of type I collagen, which can sensitively and specifically reflect human bone destruction (18). Besides, TRACP-5b in human serum is mainly secreted by osteoclasts, and is also a sensitive and specific indicator of bone destruction (19). PIEZO1 is a mechanically sensitive ion channel protein and a key force sensor for osteoblast differentiation (20). The serum level of PIEZO1 indicates bone formation (20). In this study, bone homeostasis disorders were assessed using serum CTX-I, TRACP-5b and PIEZO1 concentration. The serum CTX-I, TRACP-5b and PIEZO1 were detected by ELISA.

APACHE II scores, SOFA scores, and the combination diseases were recorded at the time of patients’ serum collection (serum was to detect bone homeostasis indicators). The operation time, IBL, intraoperative fluid balance, mechanical ventilation time, ICU stay time, hospital stay time and 30-day all caused mortality were acquired by patient records checked and discharge 30-days follow up.

Besides, blood PH value, PaCO2, HCO3- concentration, blood lactic acid concentration, white blood cell number, hemoglobin, serum creatinine, serum ALT, AST, BNP, calcium, phosphorus and albumin were all measured at the same time of patients’ serum collection (serum was to detect bone homeostasis indicators).

### Laboratory methods

Human CTX-I ELISA kit (CSB-E11224h, Cusabio, China), human TRACP-5b ELISA kit (CSB-E08490h, Cusabio, China) and human PIEZO1 ELISA kit (EH15116, FineTest, China) were used. 2 ml blood collected from patients was centrifuged at 3500 rm/r, 15 min. Then, CTX-I, TRACP-5b, and PIEZO1 of supernatant were detected according to the instructions of ELISA kit.

### Statistical analysis

SPSS statistics 25.0 (IBM, Chicago, IL, USA), MedCalc v.20.014 (MedCalc Software Ltd., Ostend, Belgium^1^; 2021) and Stata/MP 16.0 (College Station, TX77845, USA) were used to analyze the data. Shapiro-Wilk test was used to evaluate the normality of the measurement data. If the measurement data followed normal distribution, it was described as means ± standard and compared by independent-sample *t*-test between two groups or one-way ANOVA for more than two groups. If the measurement data obeyed the skewed distribution, it was represented by median (quartile range) and compared by Mann–Whitney *U* test between two group and Jonckheere-Terpstra test for more than two groups. The enumeration data was represented by occurrence rate and compared by Chi-square test or Fischer’s exact test.

Correlation coefficients were obtained by applying Spearman’s rank correlation or Pearson correlation analysis. A receiver operating characteristic (ROC) curve and precision-recall curve (PRC) were used to evaluate the correlation intensity. The optimal cutoff value was determined by the Youden index.

Logistic regression analysis was performed to find out the relationship of CTX-I, TRACP-5b, and PIEZO1 with sepsis. Clinical parameters (not included CTX-I, TRACP-5b, and PIEZO1) with *p* < 0.10 in univariate analyses were included in the multivariate logistic regression model. The multivariate logistic regression model was used to constructed the clinic prediction model. Decision curve analysis (DCA) and interventions avoided curve (IAC) were used for exploring the value of bone homeostasis disorders in the treatment of sepsis and sepsis shock. Furthermore, Kaplan–Meier curves are provided for exploring the role of bone homeostasis disorders on the mortality of sepsis and sepsis shock patients. For all analyses, *p* < 0.05 was considered statistically significant.

## Results

### Demographic and clinical characteristics of the patients

**FIGURE 1:**
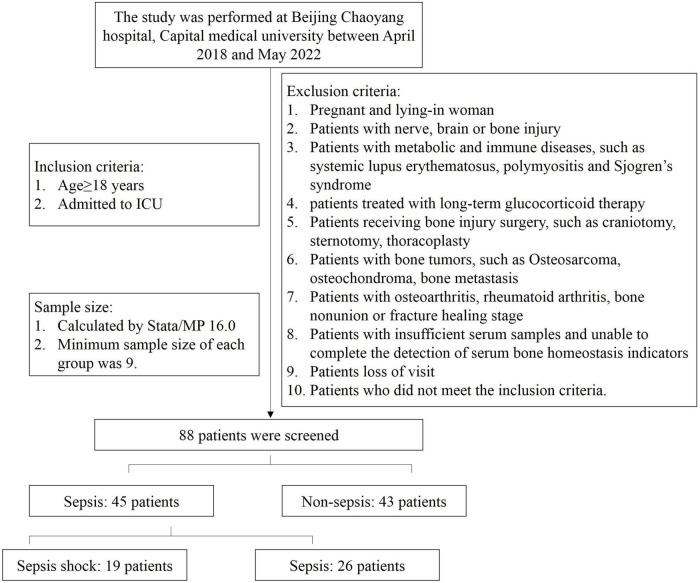
Study flow diagram.

During the study period, 88 patients were screened (Figure 1). Sepsis group were 45 patients, in additions, 29 were male and 16 were female. The age was 68 (17.5) years. In these patients, 19 were diagnosed sepsis shock. Sepsis shock patients had higher APACHE II scores, SOFA scores and longer mechanical ventilation treatment time, and higher 30-day all caused mortality. There was no significant difference in age and gender proportion among the groups, and the groups were comparable (Table 1).

**TABLE 1:** Demographics and baseline characteristics.

	Sepsis shock (*n* = 19)	Sepsis (*n* = 26)	Non-sepsis (*n* = 43)	*P*-value
Age (years)	67 (20)	68 (16)	70 (14)	0.43^a^
**Sex**				
Female	7	9	22	0.33^b^
Male	12	17	21	0.33^b^
BMI (kg/m^2^)	23.72 ± 5.11	22.82 ± 3.72	24.49 ± 3.58	0.25^c^
APACHE II score	16 (10)	11.5 (5.25)	10 (4)	<0.01^a^
SOFA score	7 (3)	3 (3)	1 (2)	<0.01^a^
**Combination diseases**				
Hypertension	11	11	22	0.57^b^
DM	3	6	11	0.70^b^
CHD	3	5	8	0.95^b^
COPD	0	3	5	0.30^b^
CLD	0	0	1	0.59^b^
CKD	2	0	1	0.14^b^
CVD	0	6	5	0.07^b^
ARDS	2	2	0	0.12^b^
AKI	3	2	0	0.04^b^
Hepatic failure	3	2	0	0.04^b^
DIC	2	1	0	0.11^b^
Postoperative patient	18	25	43	0.36^b^
Operation time (h)	3.09 (2.19)	2.25 (4.87)	3.67 (1.92)	0.22^a^
IBL	175 (275)	50 (350)	100 (150)	0.32^a^
Intraoperative fluid balance	2,450 (1,005)	1,925 (1,727.5)	1,750 (760)	0.02^a^
Mechanical ventilation (h)	26.5 (24.75)	13 (6.25)	3.33 (12)	<0.01^a^
ICU stay (h)	97 (89.25)	48.5 (59.75)	24 (21)	<0.01^a^
Hospital stay (h)	421 (667.5)	387.5 (588.25)	432 (268)	0.78^a^
30-day mortality (%)	21.05	3.85	0	<0.01^b^

^a^Jonckheere-Terpstra test.

^b^Chi-square test.

^c^One-way ANOVA (unpaired, two-tailed).

BMI, body mass index; APACHE II, acute physiology and chronic health evaluation II; SOFA, sequential organ failure assessment; DM, diabetes mellitus; CHD, coronary heart disease; COPD, chronic obstructive pulmonary disease; CLD, chronic liver disease; CKD, chronic kidney disease; CVD, cerebrovascular disease; ARDS, acute respiratory distress syndrome; AKI, acute kidney injury; DIC, disseminated intravascular coagulation; IBL, intraoperative blood loss.

### Outcomes

The results of peripheral blood indicators in three groups were showed in Table 2. Sepsis patients had lower blood PH value, higher Lactic acid level, higher ALT, AST and BNP in serum (*p* < 0.05). Besides, sepsis patients had higher serum CTX-I, TRACP-5b, PIEZO1 level (*p* < 0.05), which meant that sepsis patients had bone homeostasis disorders.

**TABLE 2:** Study outcomes.

	Sepsis shock (*n* = 19)	Sepsis (*n* = 26)	Non-sepsis (*n* = 43)	*P*-value
PH	7.42 (0.10)	7.425 (0.10)	7.45 (0.12)	0.03^a^
PaCO2 (mmHg)	37 (9.0)	36.5 (7.5)	36 (9)	0.29^a^
HCO3- (mmol/L)	25.4 (6.3)	23.75 (3.45)	25.20 (2.70)	0.23^a^
Lactic acid (mmol/L)	2.50 (2.60)	1.20 (1.57)	1.00 (0.70)	<0.01^a^
White blood cell (× 10^9^/L)	9.80 (10.50)	10.10 (8.40)	8.09 (5.10)	0.04^a^
Hemoglobin (g/L)	99.58 ± 24.11	102.88 ± 15.38	109.21 ± 15.40	0.11^b^
Creatinine (μmol/L)	65.60 (34.40)	66.20 (30.90)	55.80 (23.30)	0.21^a^
ALT (U/L)	30.00 (63.00)	30.50 (42.50)	14.00 (16.00)	<0.01^a^
AST (U/L)	48.00 (105.00)	54.50 (63.25)	21.00 (14.00)	<0.01^a^
BNP (pg/ml)	103.00 (148.00)	75.50 (71.75)	51.00 (85.00)	0.02^a^
Albumin (g/L)	25.30 (9.60)	28.40 (6.30)	31.80 (6.20)	<0.01^a^
CTX-I (ng/ml)	5.85 (7.02)	4.11 (5.29)	3.84 (4.54)	<0.01^a^
TRACP-5b (mIU/ml)	2.56 (3.00)	2.39 (1.88)	1.75 (1.45)	<0.01^a^
PIEZO1 (ng/ml)	9.37 (29.09)	5.60 (14.52)	4.60 (6.12)	0.02^a^

^a^Jonckheere-Terpstra test.

^b^One-way ANOVA (unpaired, two-tailed).

ALT, alanine aminotransferase; AST, aspartate transaminase; BNP, B-type natriuretic peptide; CTX-I, cross-linked carboxy-terminal telopeptide of type I collagen; TRACP-5b, tartrate-resistant acid phosphatase 5b; PIEZO1, piezo-type mechanosensitive ion channel component 1.

### The relation of sepsis and bone homeostasis disorders

**FIGURE 2:**
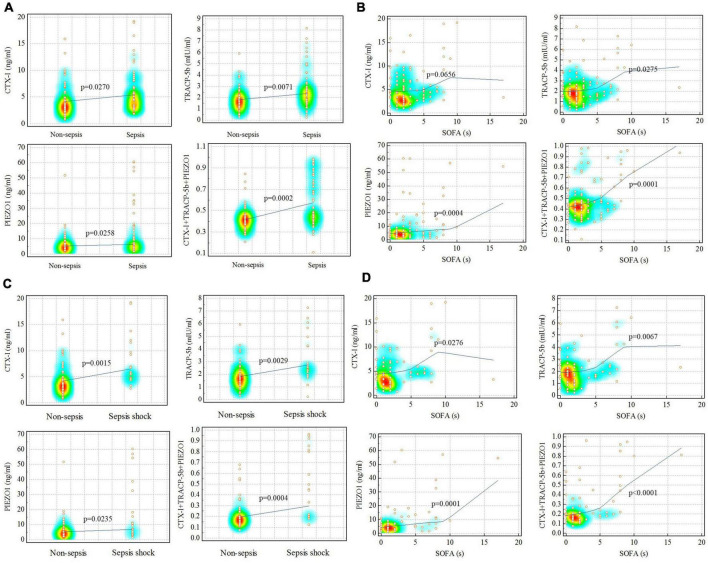
Correlation analysis for sepsis and non-sepsis. **(A,B)** Sepsis vs. non-sepsis; **(C,D)** sepsis shock vs. non-sepsis. Red represented the data concentration area.

Correlation test, ROC curve, DCA and IAC curve were used to evaluate the relation of sepsis and bone homeostasis disorders. Correlation test showed that CTX-I, TRACP-5b, PIEZO1 and the three together (CTX-I + TRACP-5b + PIEZO1) had strong correlation with sepsis (*p* = 0.03, *p* < 0.01, *p* = 0.03, *p* < 0.01, respectively). Besides, TRACP-5b, PIEZO1 and CTX-I + TRACP-5b + PIEZO1 had also strong correlation with SOFA scores (*p* = 0.03, *p* < 0.01, *p* < 0.01, respectively; Figure 2).

**FIGURE 3:**
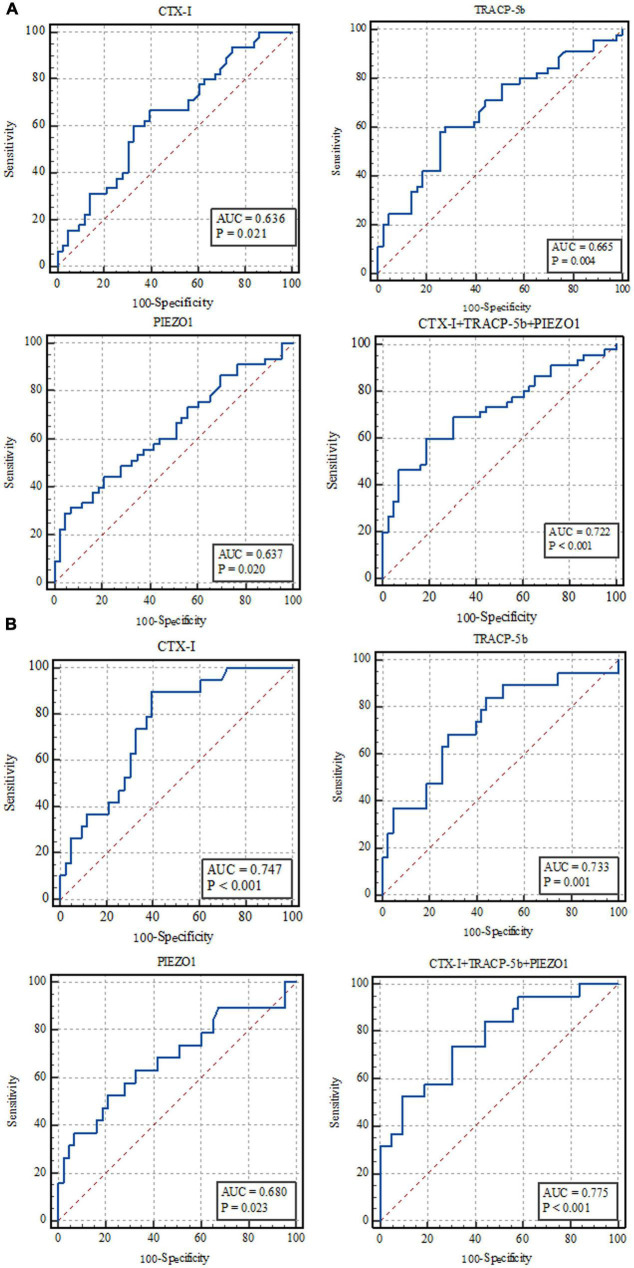
ROC analysis results. **(A)** Sepsis vs. non-sepsis; **(B)** sepsis shock vs. non-sepsis.

ROC curve results showed that these indicators could predict the occurrence of sepsis (*p* = 0.02, *p* < 0.01, *p* = 0.02, *p* < 0.01, respectively; Figure 3). Area under the ROC curve (AUC) were 0.636, 0.665, 0.637, and 0.722, which further confirmed the correlation test results. The PRC results further confirmed the ROC results (details in Supplementary material).

**FIGURE 4:**
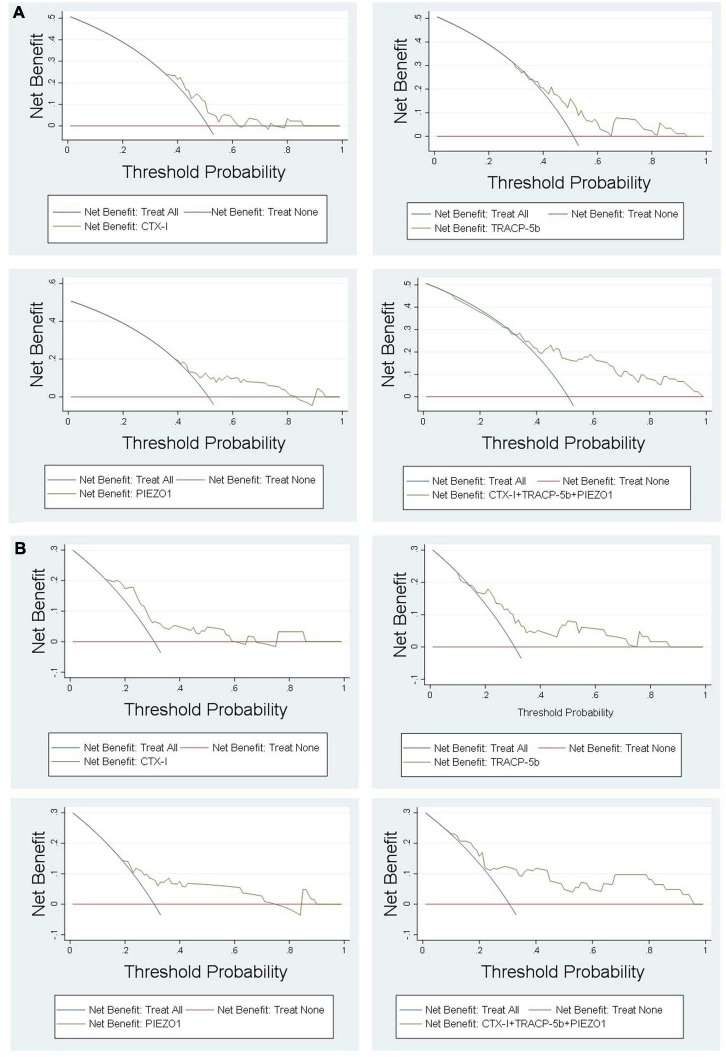
DCA results. **(A)** Sepsis vs. non-sepsis; **(B)** sepsis shock vs. non-sepsis.

**FIGURE 5:**
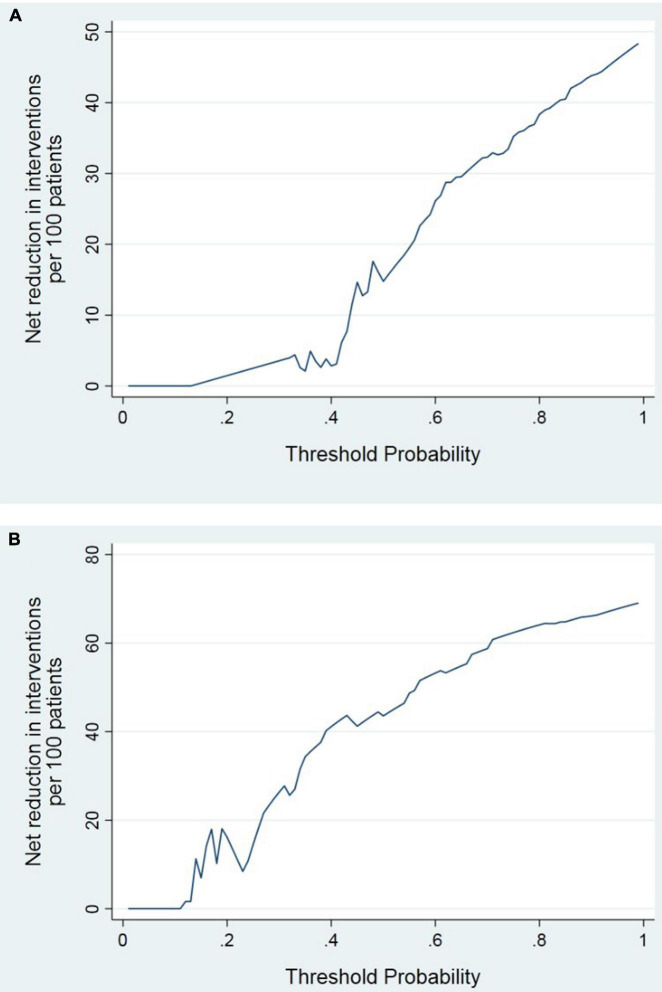
IAC analysis results. **(A)** Sepsis vs. non-sepsis; **(B)** sepsis shock vs. non-sepsis.

DCA curve showed the value of bone homeostasis disorders in the treatment of sepsis (Figure 4). The X-axis indicates the threshold probability for sepsis development and the Y-axis indicates the net benefit. A high net benefit was provided by those indicators models that are far away from the slanted dash blue line and the horizontal red line (Figure 4). IAC results showed the treatment value of bone homeostasis disorders in sepsis. The X-axis also indicates the threshold probability for sepsis development and the Y-axis indicates the net reduction in intervention per 100 patients. The result showed that sepsis at 60% of the probability threshold, bone homeostasis indicators could reduce nearly 25% patients using the existing diagnosis means in clinic, including invasive operation inspection (Figure 5).

### The relation of sepsis shock and bone homeostasis disorders

The correlation between sepsis shock and bone homeostasis disorders was also assessed. Correlation test showed that CTX-I, TRACP-5b, PIEZO1 and the three together (CTX-I + TRACP-5b + PIEZO1) had strong correlation with sepsis shock (*p* < 0.01, *p* < 0.01, *p* = 0.02, *p* < 0.01, respectively) and SOFA (*p* = 0.03, *p* < 0.01, *p* < 0.01, *p* < 0.01, respectively; Figure 2).

ROC curve results further confirmed the correlation test results, which showed that these indicators could predict the sepsis shock occurrence (*p* < 0.01, *p* < 0.01, *p* = 0.02, *p* < 0.01, respectively; Figure 3). The AUC were 0.747, 0.733, 0.680, and 0.775. The PRC results further confirmed the ROC results (details in Supplementary material).

DCA showed that the high net benefit was provided by those indicators models that are far away from the slanted dash blue line and the horizontal red line (Figure 4). IAC results showed that the bone homeostasis indicators could reduce almost 40% patients using the existing diagnosis means in clinic, including invasive operation inspection, when sepsis shock at 40% of the probability threshold (Figure 5).

### Bone homeostasis disorders and the mortality of sepsis shock

**FIGURE 6:**
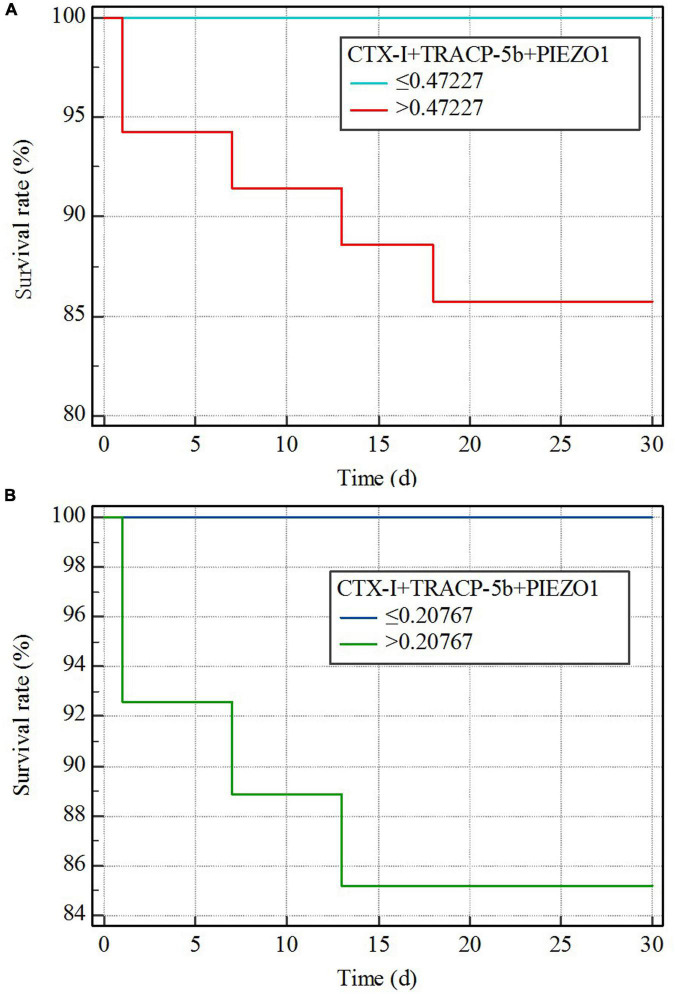
Kaplan–Meier curves. **(A)** Sepsis vs. non-sepsis; **(B)** sepsis shock vs. non-sepsis.

Kaplan–Meier curves are explored the relationship between bone homeostasis disorders and the mortality of sepsis, sepsis shock. The Youden index was used to calculate the optimal cutoff value. The cutoff value of the three indicators together (CTX-I + TRACP-5b + PIEZO1) on sepsis prediction was 0.47227 and on sepsis shock prediction was 0.27256. Then, sepsis or sepsis shock patients were divided into two groups based on the value of the three indicators together. Kaplan–Meier curves showed the mortality difference of the two groups. Sepsis patients with high value (>the cutoff value of the three indicators together) had the higher mortality (*p* < 0.01), besides, the curve showed a higher mortality of sepsis shock patients with high value (>the cutoff value of the three indicators together) (*p* = 0.02; Figure 6).

## Discussion

Bone tissue is rich in nerve and blood vessels. In recent years, it has been found that bone tissue has a regulatory effect on various organs of the body, such as brain, kidney, and liver (21–23). Besides, bone played a very important role on immune functions (14–16). Bone homeostasis reflects immune system reaction abilities. Sepsis causes organ dysfunction after infection, which is closely related to body immune system. So far, there is no specific effective drugs on sepsis treatment, and nearly 20% of all deaths are sepsis related (3). To find out the influence factors of sepsis occurrence and development, so as to provide a research basis for finding drugs for the treatment of sepsis or early diagnosis. At present, there is no research on the relationship between bone homeostasis and sepsis. This study was used retrospective cohort study method to preliminarily explore the relationship between bone homeostasis and sepsis.

CTX-I, TRACP-5b, and PIEZO1 were used as indicators to evaluate bone homeostasis disorders. First, comparing sepsis group with non-sepsis group, it was found that CTX-I, TRACP-5b, and PIEZO1 in sepsis group all were significantly higher than that in non-sepsis group, which indicated that sepsis could lead to bone homeostasis disorders. At the same time, the CTX-I, TRACP-5b, and PIEZO1 of sepsis shock group were also higher than that of sepsis group, indicating that the disorders of bone homeostasis increased as the aggravation of sepsis.

CTX-I is the degradation product of type I collagen. Collagen in bone tissue is mainly type I collagen. This study found that sepsis is associated with CTX-I, indicating that sepsis may be closely related to type I collagen metabolism. At present, there are few literatures on the correlation between collagen metabolism and sepsis. Gäddnäs et al. (24) found that collagen degradation was associated with sepsis in 2009. However, type I and type II were not clear (25). TRACP-5b is a cytokine mainly secreted by osteoclasts, which can sensitively and specifically reflect the activity of osteoclasts in the body. Our study found that elevated TRACP-5b could predict sepsis occurrence and was closely related to sepsis mortality. At present, only a few studies have found a possible relationship between osteoclast function and sepsis through transcriptomics (26, 27). This study provided a basis for exploring the relationship between osteoclasts and sepsis. PIEZO1 is a mechanically sensitive protein and is closely related to osteoblast differentiation. At present, there are few studies on PIEZO1 and sepsis. Aykut et al. (28) proposed that PIEZO1 might be related to sepsis. Inhibition of PIEZO1 had a positive effect on the treatment of sepsis. However, the specific mechanism was needed the further study.

Since there were differences in bone homeostasis indicators among sepsis, sepsis shock and non-sepsis groups, correlation was used to evaluate whether there was a relationship between bone homeostasis and sepsis, sepsis shock. The results showed that there was a strong correlation between bone homeostasis disorders and sepsis, sepsis shock. Z.A. Puthucheary et al. (29) investigated the relationship between sepsis and bone. They used rat sepsis model induced by cecal ligation and puncture (CLP) and found that rat femoral trabecular bone strength was reduced within 24 h and was associated with collagen reduction. J. Bayer et al. (30) also used animal CLP induced sepsis model and proposed that the bone was the major source of high circulating intact fibroblast growth factor-23 in asepsis animal. A. Terashima (31) reviewed the role of bone cells in immune regulation during the course of infection and summarized that bone homeostasis played a crucial role in infection. However, few clinical studies explored the relationship between bone homeostasis and sepsis, sepsis shock. Moreover, whether bone homeostasis disorders aggravated sepsis severity had not been discussed.

In addition to the strong correlation between bone homeostasis and sepsis, we also used bone homeostasis indicators (CTX-I, TRACP-5b, and PIEZO1) to predict sepsis and sepsis shock. The ROC and PRC results showed that bone homeostasis indicators could predict the occurrence of sepsis and sepsis shock, which is a surprising result. Besides, the prediction efficiency was above 0.5. At present, there was no reports about the prediction of bone homeostasis on sepsis. DCA and IAC results confirmed the ROC results, which showed a high net benefit provided by bone homeostasis indicators on sepsis or sepsis shock.

Since bone homeostasis could predict sepsis and sepsis shock, we further evaluated the relationship between bone homeostasis and mortality of sepsis or sepsis shock. The Youden index was used to calculate the optimal cutoff value and the cutoff value was used to divided group. We found that patients with bone homeostasis indicators exceeded the cutoff value had higher mortality, both in sepsis and sepsis shock patients. This indicated that bone homeostasis disorders could aggravate the illness degree of sepsis patients.

The innovation of this study was obvious. First of all, we found that sepsis and sepsis shock were related to bone homeostasis disorders through clinical observation. Secondly, we used a variety of statistical methods, including DCA, IAC, PRC, and found that bone homeostasis indicators could predict the occurrence of sepsis and sepsis shock. Finally, we found that bone homeostasis disorders could increase the mortality of sepsis and sepsis shock patients through Kaplan–Meier curve.

The limitations of this study were also obvious. Firstly, although we calculated the sample size, the sample size of this study was still small. Secondly, this study was a retrospective study, not a prospective cohort study. Besides, this study included a single center and we planned to carry out a multicenter prospective cohort study in future. Thirdly, because this study did not use external data including public databases to validate, the results have limitations. Besides, there was no significant difference in age, gender, BMI, combination diseases between sepsis and non-sepsis group, which might be the result of limited sample size. Fourthly, the bone homeostasis biomarkers could be time-varying in a longitudinal dataset, which was not currently captured (32). In addition, the causal relationship between these bone homeostasis biomarkers and sepsis outcomes was not clear. This was required animal experiments or cell experiments to explore the relationship. Finally, we intended to explore the regulatory mechanism between bone homeostasis and sepsis by animal or cell experiments, so as to provide a research basis for the treatment of sepsis.

## Conclusion

Bone homeostasis was closely related to the occurrence of sepsis and sepsis shock. The indicators, such as CTX-I, TRACP-5b, and PIEZO1, could predict the occurrence of sepsis and sepsis shock. Using these indicators to predict sepsis could get good net benefit. In additions, bone homeostasis disorders could increase the mortality of sepsis and sepsis shock patients, which were a notable problem.

## Data availability statement

The original contributions presented in this study are included in the article/Supplementary material, further inquiries can be directed to the corresponding authors.

## Ethics statement

The studies involving human participants were reviewed and approved by Beijing Chaoyang Hospital of Capital Medical University. The patients/participants provided their written informed consent to participate in this study.

## Author contributions

All authors made a significant contribution to the work reported, whether that is in the conception, study design, execution, acquisition of data, analysis and interpretation, or in all these areas; took part in drafting, revising or critically reviewing the article; gave final approval of the version to be published; have agreed on the journal to which the article has been submitted; and agree to be accountable for all aspects of the work.

## Footnotes

1
https://www.medcalc.org
