# Supplementary material for: Bone homeostasis disorders increased the mortality of sepsis patients: A preliminary retrospective cohort study
Source: Front Med (Lausanne). 2022 Dec 1;9:1017411. doi: 10.3389/fmed.2022.1017411 (PMC9751061; doi:10.3389/fmed.2022.1017411)
Supplement: Supplementary file 1 [file Data_Sheet_1.DOCX]

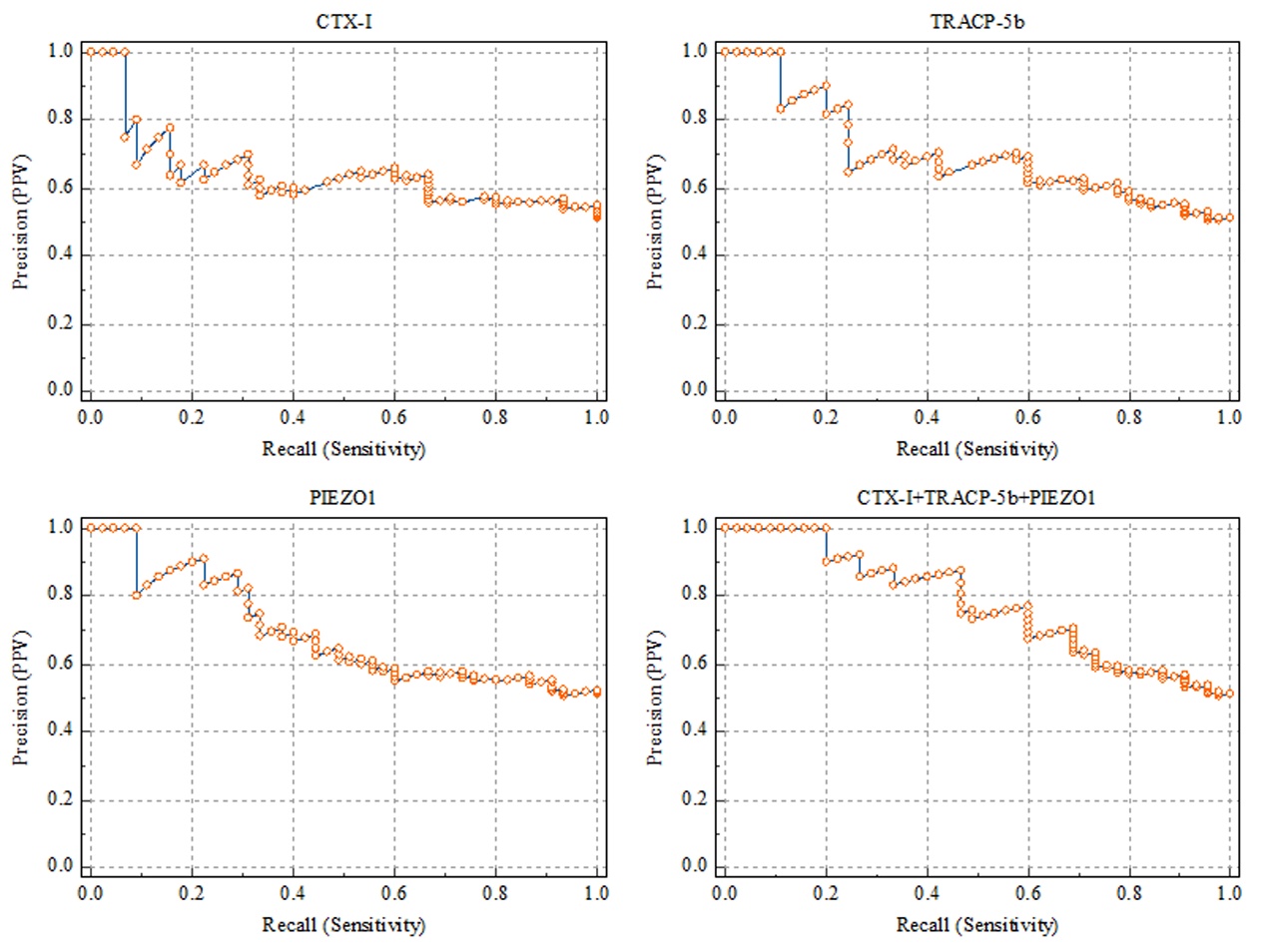


Fig.1 The PRC results of sepsis compared with non-sepsis. The area under curve were all above 0.5.


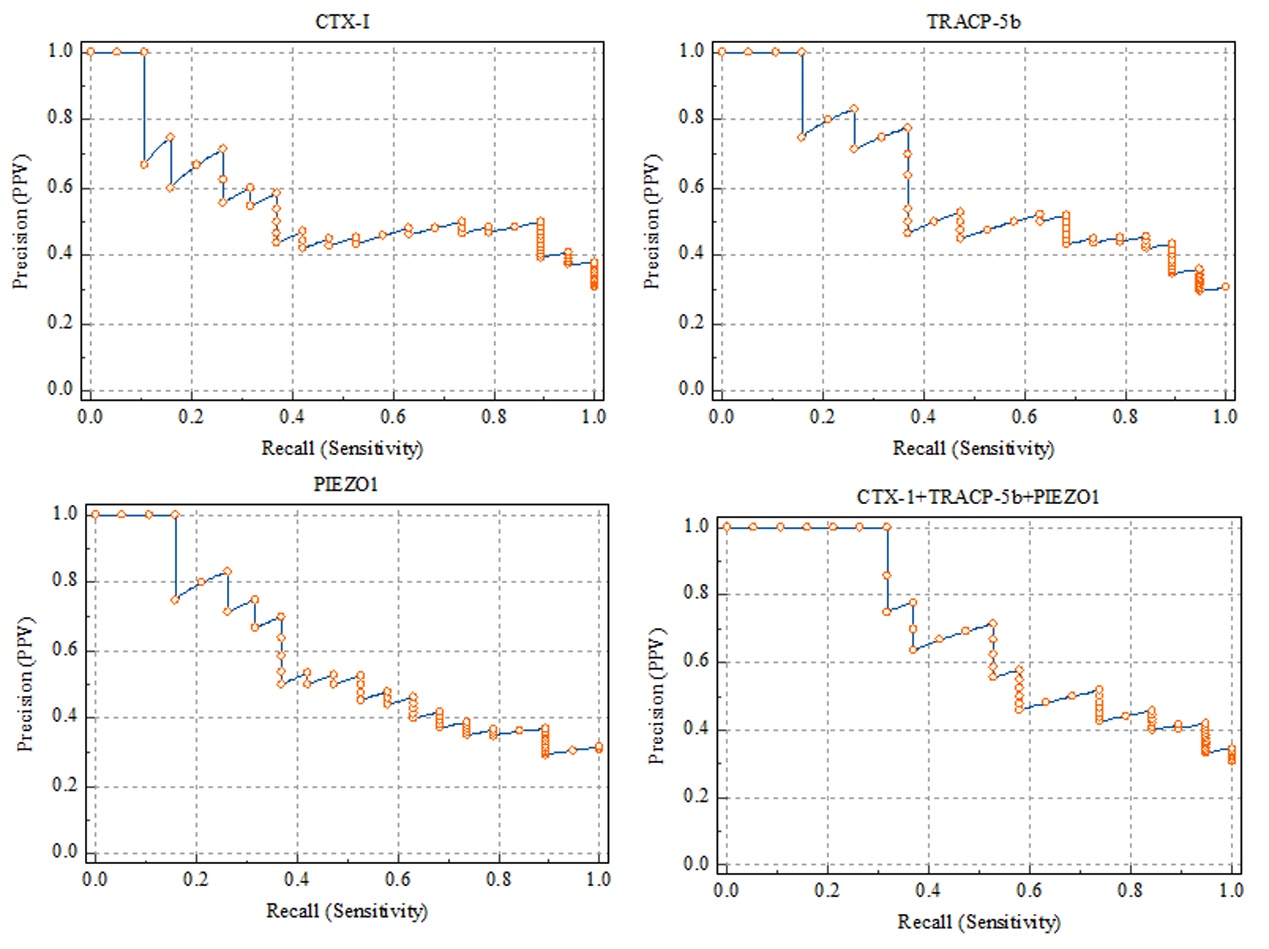


Fig.2 The PRC results of sepsis shock compared with non-sepsis. The area under curve were all above 0.5.
